# Stent thrombosis associated with drug eluting stents on addition of cilostazol to the standard dual antiplatelet therapy following percutaneous coronary intervention: a systematic review and meta-analysis of published randomized controlled trials

**DOI:** 10.1186/s40360-018-0224-3

**Published:** 2018-06-18

**Authors:** Feng Huang

**Affiliations:** Institute of Cardiovascular Diseases and Guangxi Key Laboratory Base of Precision Medicine in Cardio-cerebrovascular Diseases Control and Prevention, The First Affiliated Hospital of Guangxi Medical University, Nanning, Guangxi 530021 P. R. China

**Keywords:** Dual antiplatelet therapy, Triple antiplatelet therapy, Cilostazol, Percutaneous coronary intervention, Stent thrombosis

## Abstract

**Background:**

In this analysis, we aimed to systematically compare stent thrombosis (ST) and its different subtypes following treatment with DAPT (aspirin + clopidogrel) versus TAPT (aspirin + clopidogrel + cilostazol).

**Methods:**

Studies were included if: they were randomized controlled trials (RCTs) comparing TAPT (cilostazol + aspirin + clopidogrel) with DAPT (aspirin + clopidogrel); they reported ST or its subtype including definite, probable, acute, sub-acute and late ST as their clinical outcomes. RevMan software (version 5.3) was used to carry out this analysis whereby odds ratios (OR) and 95% confidence intervals (CI) were generated.

**Results:**

Statistical analysis of the data showed no significant difference in total ST with the addition of cilostazol to the standard DAPT with OR: 0.65, 95% CI: 0.38–1.10; *P* = 0.11, I^2^ = 6%. Moreover, when ST was further subdivided and analyzed, still, no significant difference was observed in acute, sub-acute, late, definite and probable ST with OR: 0.48, 95% CI: 0.13–1.74; *P* = 0.27, I^2^ = 0%, OR: 0.56, 95% CI: 0.22–1.40; *P* = 0.21, I^2^ = 0%, OR: 0.72, 95% CI: 0.23–2.28; *P* = 0.58, I^2^ = 0%, OR: 1.18, 95% CI: 0.38–3.69; *P* = 0.77, I^2^ = 3% and OR: 0.75, 95% CI: 0.17–3.55; *P* = 0.70, I^2^ = 0% respectively. No change was observed during a short term (≤ 6 months) and a longer (≥ 1 year) follow-up time period.

**Conclusions:**

This current analysis showed no significant difference in stent thrombosis with the addition of cilostazol to the standard dual antiplatelet therapy during any follow-up time period after PCI.

## Background

Nowadays, percutaneous coronary intervention (PCI) is mainly carried out with drug eluting stents (DES). In the year 2017, a clinically interesting meta-analysis of randomized controlled trials showed similar cardiovascular outcomes in patients who were discharged on the same day versus patients who stayed overnight in the hospital following PCI [1]. However, the main shortcoming of DES is the occurrence of stent thrombosis (ST) [2].

In order to minimize ST, the 2014 European Society of Cardiology (ESC) and the European Association of Percutaneous Cardiovascular Interventions (EAPCI) guidelines on myocardial revascularization recommend the use of dual antiplatelet therapy (DAPT) consisting of aspirin and clopidogrel for at least six months in patients with stable coronary artery disease and for at least one year in patients with acute coronary syndrome [3]. However, recent progress in clinical medicine showed the addition of cilostazol (another antiplatelet agent) to DAPT, now called triple antiplatelet therapy (TAPT), to be more effective in comparison to DAPT [4] especially in decreasing repeated revascularization.

Further updated meta-analyses compared the outcomes which were associated with DAPT (aspirin + clopidogrel) and TAPT (cilostazol + aspirin + clopidogrel) [5, 6]. However, ST was never well compared systematically.

In contrast to other previously published meta-analyses, we aimed to systematically compare ST and its different subtypes following treatment with DAPT (aspirin + clopidogrel) versus TAPT (aspirin + clopidogrel + cilostazol) to show any significant difference related to ST.

## Methods

### Searched databases

The following databases were searched:

1. The Cochrane database;
2. EMBASE (www.sciencedirect.com);
3. MEDLINE;
4. www.ClinicalTrials.gov;
5. Reference lists of relevant publications.

### Searched terms

The following terms were searched:

1. Dual antiplatelet therapy versus triple antiplatelet therapy;
2. Cilostazol and percutaneous coronary intervention;
3. Cilostazol and coronary angioplasty;
4. Cilostazol, aspirin and clopidogrel;
5. Triple antiplatelet therapy and percutaneous coronary intervention;
6. DAPT versus TAPT;
7. DAPT versus cilostazol.

### Inclusion criteria

Studies were included if:

1. They were randomized controlled trials (RCTs) comparing TAPT (cilostazol + aspirin + clopidogrel) with DAPT (aspirin + clopidogrel);
2. They reported ST (or its subtype including definite, probable, acute, sub-acute and late ST) as their clinical outcomes.

### Exclusion criteria

Studies were excluded if:

1. They were meta-analyses, review articles, observational cohorts, case-control studies and letter to editors;
2. TAPT did not consist of cilostazol, but instead, consisted of another antiplatelet or antithrombotic drug such as warfarin;
3. ST was not reported among the clinical outcomes;
4. They were duplicated studies.

### Type of patients, outcomes, definitions and follow-ups

Several types of patients with CAD who were treated by PCI were included in this analysis (Table 1):

1. Patients with type 2 diabetes mellitus (T2DM);
2. Patients with obesity;
3. Patients with acute coronary syndrome (ACS);
4. Patients with long coronary lesions (LCL);
5. Patients with coronary bifurcation;
6. Patients with native CAD;
7. Patients with multi-vessel CAD.

**Table 1:** Types of stent thrombosis which were reported

Studies	Type of stent thrombosis reported	Follow-up period	Type of participants	Type of stent
Ahn2008 [10]	Acute, sub-acute and late ST	6 months	PCI in patients with T2DM	DES
Gao2013 [11]	Definite, probable, acute and late ST	1 year	PCI in patients with obesity	DES
Han2009 [12]	Sub-acute ST	1 month	PCI in patients with ACS	DES
Lee2005 [13]	Acute and sub-acute ST	1 month	PCI in patients with CAD	DES
Lee2010A [14]	Acute, sub-acute, late and very late ST	2 years	PCI in patients with T2DM and LCL	DES
Lee2011 [15]	Acute, sub-acute, late ST	1 year	PCI in patients with LCL	DES
Suh2011 [16]	ST	6 months	PCI in patients with native CAD	DES
Youn2014 [17]	ST, definite and probable ST	3 months and 1 year	PCI in patients with LCL or MVD	DES
Zhu2015 [18]	Sub-acute and late ST	1 year	PCI in patients with ACS	DES
Park2013 [19]	Definite and probable ST	1 month	PCI in patients with CAD	DES

Abbreviations: ST: Stent thrombosis, PCI: Percutaneous coronary intervention, T2DM = type 2 diabetes mellitus, ACS: Acute coronary syndrome, CAD: Coronary artery disease, LCL: Long coronary lesions, MVD: Multi-vessel diseases, DES: Drug eluting stents

ST and its subtypes including (Table 1):

1. Total ST: the total number of any type of ST;
2. Acute ST: less than 1 day;
3. Sub-acute ST: 1 day to 1 month;
4. Late ST: 1 to 12 months or more;
5. Definite ST and;
6. Probable ST were assessed.

Definite and probable ST were defined according to the Academic Research Consortium [7].

The follow-up time periods were as followed:

1. A short term follow up period of 6 months or less.
2. A longer follow up time period of 1 year or more (1–3 years) as shown in Table 1.

### Data extraction and quality assessment

The following data were extracted and cross-checked by the reviewer Feng Huang:

1. The type of study (trial or observational cohort);
2. The total number of patients who were treated by DAPT and TAPT respectively;
3. The types of participants;
4. The patients’ enrollment time periods;
5. The baseline characteristics of the participants;
6. The follow-up time periods.

Another reviewer (Pravesh Kumar Bundhun) was also involved in the searched process and in data extraction. However, because he did not satisfy all the criteria for authorship, he was only acknowledged at the end of the paper.

The methodological quality was assessed in accordance to the criteria suggested by the Cochrane collaboration (for randomized controlled trials) [8]. Grades were allotted (A to E with a grade A implying a low risk of bias).

### Statistical analysis

RevMan analytical software for meta-analysis (version 5.3) was used to carry out this analysis whereby odds ratios (OR) and 95% confidence intervals (CI) were generated.

Heterogeneity was assessed by two simple methods:

1. The Q statistic test whereby a *P* value less or equal to 0.05 was considered statistically significant;
2. The I^2^ statistic test which focused on the value of I^2^ (the greater the value, the higher the heterogeneity).

In addition, a fixed effects model (I^2^ < 50%) or a random effects model (I^2^ > 50%) was used based on the I^2^ value which was obtained.

Sensitivity analysis was also carried out by an exclusion method (each trial was excluded one by one and a new analysis was carried out each time and the results were observed for any significant difference).In addition, publication bias was visually estimated through funnel plots.

Since registration for meta-analyses was not compulsory, protocol for this study was not prospectively registered.

### Ethics

Ethical approval was not required for such types of research articles.

## Results

### Searched outcomes

The PRISMA guideline was followed [9]. This search resulted in a total number of 788 articles. Six hundred and ninety-five (695) articles were eliminated since they were not related to this research title. Ninety three (93) full text articles were assessed for eligibility. Further elimination was carried out due to the following reasons:

1. They were meta-analyses (14);
2. They were observational studies (3);
3. They were letters to editors (3);
4. They reported platelet aggregation as outcomes (8);
5. They did not report ST among the cardiovascular outcomes (5);
6. They involved another drug in the triple antiplatelet group (23);
7. They were duplicated studies (27).

**Fig. 1:**
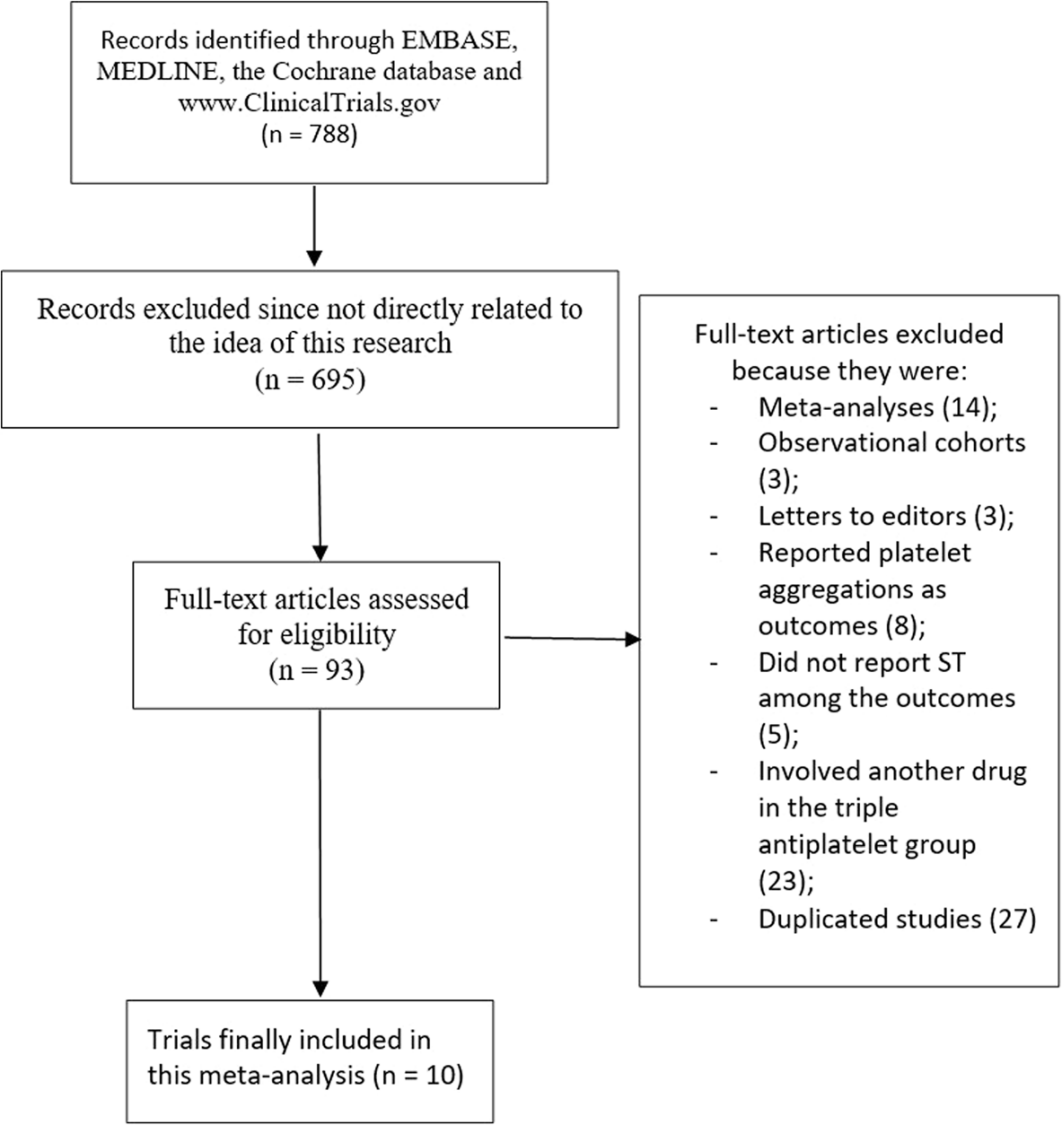
Flow diagram representing the study selection

Finally 10 randomized controlled trials [10–19] were confirmed for this analysis as shown in Fig. 1.

### General features of the studies which were included

The general features of the studies have been listed in Table 2. Ten randomized controlled trials consisting of a total number of 11, 878 participants (6035 patients were assigned to the DAPT group and 5843 patients were assigned to the TAPT group). The time period for patients’ enrollment varied from years 1998 to 2011. A detailed data set for the total number of patients which were extracted from each trial has been shown in Table 2.

**Table 2:** General features of the studies which were included

Studies	No of patients with DAPT (n)	No of patients with TAPT (n)	Type of study	Year of patients’ enrollment	Bias risk grade
Ahn2008 [10]	124	113	RCT	2004–2006	B
Gao2013 [11]	215	213	RCT	–	B
Han2009 [12]	608	604	RCT	–	B
Lee2005 [13]	1597	1415	OS	1998–2003	–
Lee2010A [14]	450	450	RCT	2004–2006	A
Lee2011 [15]	249	250	RCT	2007–2008	A
Suh2011 [16]	458	457	RCT	2006–2009	A
Youn2014 [17]	307	308	RCT	2010–2011	B
Zhu2015 [18]	151	154	RCT	–	B
Park2013 [19]	1876	1879	RCT	2010–2011	B
Total (n)	6035	5843			

Abbreviations: DAPT: Dual anti-platelet therapy, TAPT: Triple antiplatelet therapy, RCT: Randomized controlled trials, OS: Observational studies

As previously stated, the bias risk was assessed in accordance to the criteria suggested by the Cochrane collaboration. A grade ‘A’ with low risk bias was allotted to three randomized trials, whereas a grade ‘B’ was allotted to the other remaining 6 trials.

### Baseline characteristics of the participants

The baseline features of the participants have been listed in Table 3. The participants had a mean age ranging from 55.3 to 65.0 years. In addition, male patients were predominant in both groups (DAPT and TAPT). Other co-morbidities or risk factors such as hypertension, dyslipidemia, diabetes mellitus and current smoker were also reported in Table 3. According to the data which were presented, no significant difference was observed in the baseline features among those participants who were assigned to the DAPT or TAPT groups.

**Table 3:** Baseline features of the studies which were included

Studies	Age (years)	Males (%)	HT (%)	Ds (%)	DM (%)	Cs (%)
	DT/TT	DT/TT	DT/TT	DT/TT	DT/TT	DT/TT
Ahn2008 [10]	62.0/61.2	54.7/61.7	54.0/48.9	25.2/19.1	100/100	34.5/39.5
Gao2013 [11]	55.3/57.6	81.9/78.9	54.4/56.3	21.4/24.9	16.2/19.2	42.3/38.5
Han2009 [12]	60.2/59.6	72.9/73.8	56.1/57.9	45.4/45.5	20.1/23.3	–
Lee2005 [13]	59.0/59.0	71.8/71.8	46.1/42.3	27.6/26.8	26.2/23.4	31.9/32.4
Lee2010A [14]	61.0/60.9	60.7/62.2	57.1/57.0	28.5/30.2	62.4/63.3	34.7/31.6
Lee2011 [15]	62.1/60.9	71.5/70.0	64.7/58.4	45.0/42.4	33.7/36.8	30.1/30.4
Suh2011 [16]	64.0/64.8	68.3/68.6	66.6/64.5	–	32.2/35.5	26.8/23.7
Youn2014 [17]	64.2/65.0	64.2/63.0	65.8/68.2	47.6/49.4	30.9/32.5	44.0/48.4
Zhu2015 [18]	60.1/60.2	64.9/66.9	45.7/41.6	57.0/51.3	21.9/17.5	32.5/39.0
Park2013 [19]	63.7/62.8	67.0/69.8	68.6/66.8	62.7/64.2	31.3/31.8	30.8/32.8

Abbreviations: DT: Dual antiplatelet therapy, TT: Triple antiplatelet therapy, HT: Hypertension, ds: Dyslipidemia, DM: Diabetes mellitus, Cs: Current smoker

### Main results of this analysis

Results of this analysis have been represented in Table 4.

**Table 4:** Results of this analysis

Outcomes	OR with 95% CI	P value	I^2^ (%)	Statistical model used
ST	0.65 [0.38–1.10]	0.11	6	Fixed effects
Definite ST	1.18 [0.38–3.69]	0.77	3	Fixed effects
Probable ST	0.75 [0.17–3.35]	0.70	0	Fixed effects
Acute ST	0.48 [0.13–1.74]	0.27	0	Fixed effects
Sub-acute ST	0.56 [0.22–1.40]	0.21	0	Fixed effects
Late ST	0.72 [0.23–2.28]	0.58	0	Fixed effects

Abbreviations: OR: Odds ratios, CI: Confidence intervals, ST: Stent thrombosis, RCT: Randomized controlled trials, OS: Observational studies

**Fig. 2:**
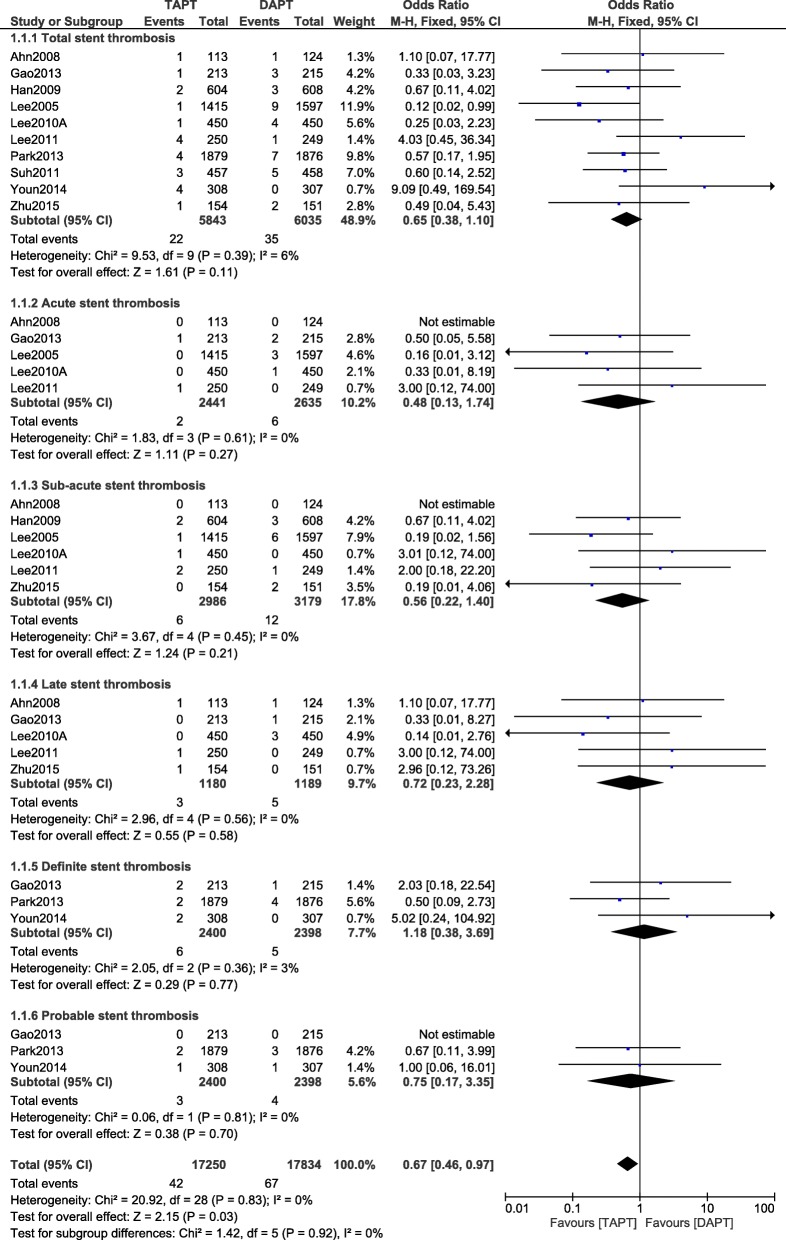
Stent thrombosis observed with the addition of cilostazol to the standard DAPT

Statistical analysis of the data showed no significant difference in total ST with the addition of cilostazol to the standard DAPT with OR: 0.65, 95% CI: 0.38–1.10; *P* = 0.11, I^2^ = 6% as shown in Fig. 2.

When ST was further subdivided and analyzed, still, no significant difference was observed in acute, sub-acute, late, definite and probable ST with OR: 0.48, 95% CI: 0.13–1.74; *P* = 0.27, I^2^ = 0%, OR: 0.56, 95% CI: 0.22–1.40; *P* = 0.21, I^2^ = 0%, OR: 0.72, 95% CI: 0.23–2.28; *P* = 0.58, I^2^ = 0%, OR: 1.18, 95% CI: 0.38–3.69; *P* = 0.77, I^2^ = 3% and OR: 0.75, 95% CI: 0.17–3.35; *P* = 0.70, I^2^ = 0% respectively as shown in Fig. 2.

Another analysis was carried out based on the follow-up time period.

**Fig. 3:**
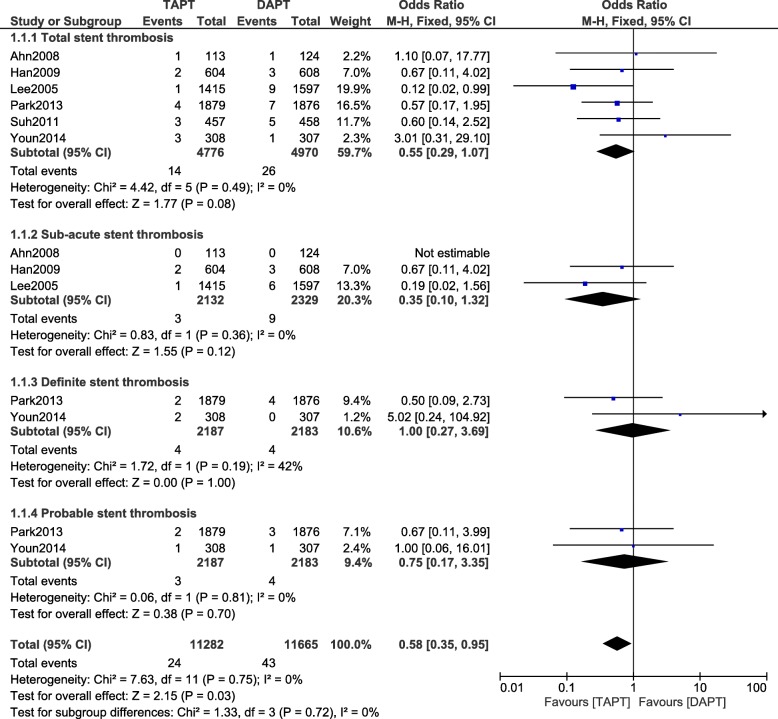
Stent thrombosis observed with the addition of cilostazol to the standard DAPT during a short follow-up time period

During a short term follow-up time period, total, sub-acute, definite and probable ST were again similarly manifested with OR: 0.55, 95% CI: 0.29–1.07; *P* = 0.08, I^2^ = 0%, OR: 0.35, 95% CI: 0.10–1.32; *P* = 0.12, I^2^ = 0%, OR: 1.00, 95% CI: 0.27–3.69; *P* = 1.00, I^2^ = 42% and OR: 0.75, 95% CI: 0.17–3.35; P = 0.70, I^2^ = 0% respectively as shown in Fig. 3.

**Fig. 4:**
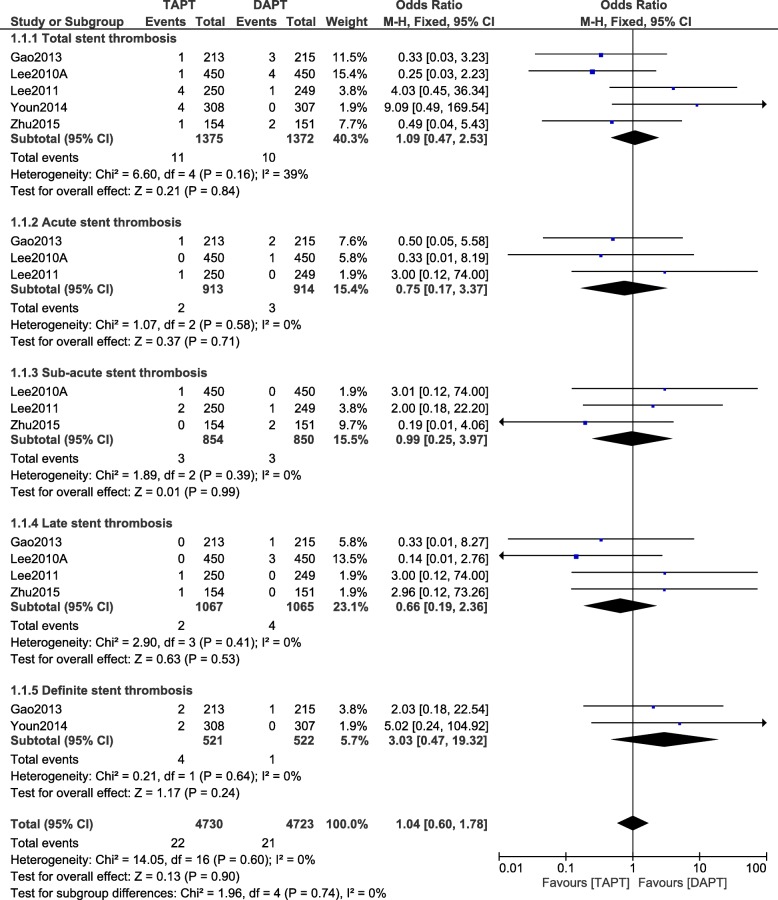
Stent thrombosis observed with the addition of cilostazol to the standard DAPT during a longer follow-up time period

During a longer follow-up time period, still no significant difference was observed in total, acute, sub-acute, late, and definite ST with the addition of cilostazol to the standard DAPT, with OR: 1.09, 95% CI: 0.47–2.53; *P* = 0.84, I^2^ = 39%, OR: 0.75, 95% CI: 0.17–3.37; *P* = 0.71, I^2^ = 0%, OR: 0.99, 95% CI: 0.25–3.97; *P* = 0.99, I^2^ = 0%, OR: 0.66, 95% CI: 0.19–2.36; *P* = 0.53, I^2^ = 0%, and OR: 3.03, 95% CI: 0.47–19.32; *P* = 0.24, I^2^ = 0% respectively as shown in Fig. 4.

Sensitivity analysis was also carried out. No significant difference in results were obtained when each study was excluded one by one.

**Fig. 5:**
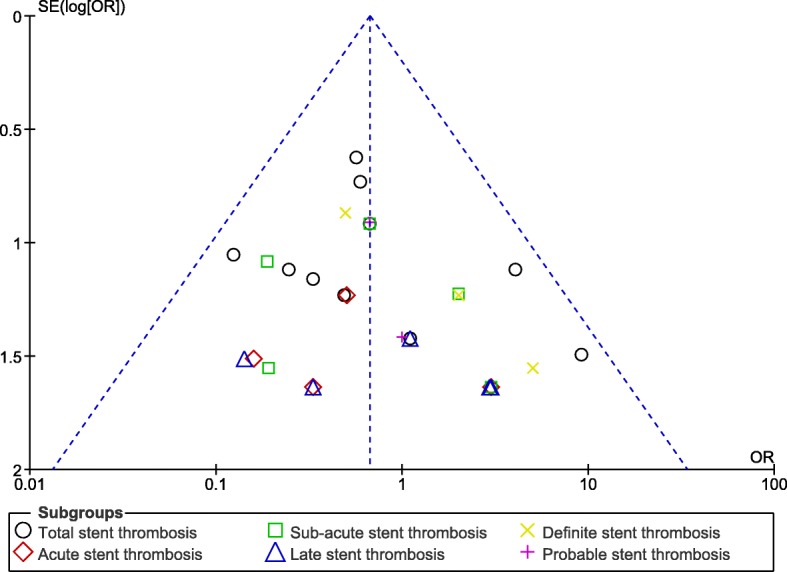
Funnel plot showing publication bias (A)

**Fig. 6:**
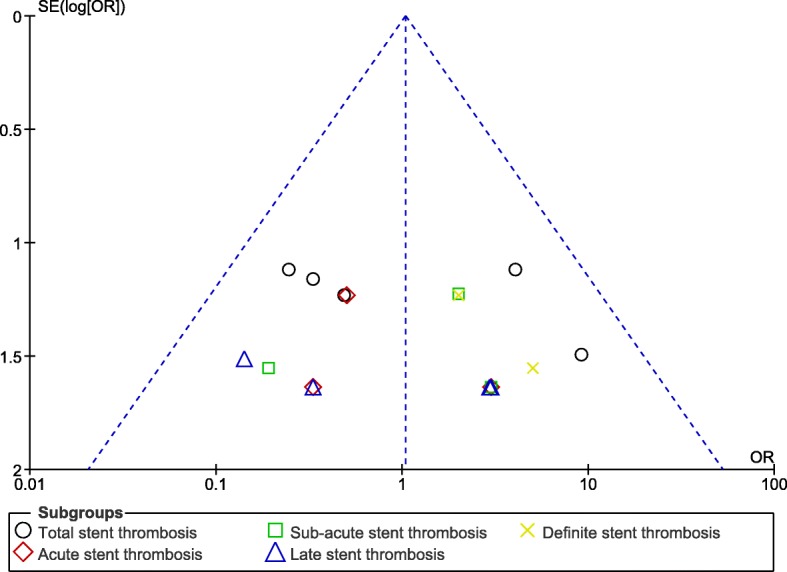
Funnel plot showing publication bias (B)

Since this analysis consisted of a small volume of studies, publication bias could better be represented by funnel plots. After carefully assessing the funnel plots, no evidence of publication bias was observed across all the trials which assessed the different subtypes of ST in this analysis as shown in Figs. 5 and 6.

## Discussion

Even though the ESC/EACTS guidelines recommend DAPT as the treatment of choice following PCI with DES, we aimed to show whether the addition of cilostazol to DAPT might potentially be associated with significantly lower ST.

In this analysis, the addition of cilostazol to the standard DAPT (aspirin and clopidogrel) did not show any significant difference in total ST or any of its subtypes including acute, sub-acute, late, definite and probable ST. No significant difference was observed even during a short (≤ 6 months) or a longer follow-up time period (≥ 1 year) after PCI.

In 2015, a clinically important meta-analysis which was published in BMC Cardiovascular Disorders compared DAPT with TAPT (cilostazol + aspirin + clopidogrel) in patients with T2DM. In their results, the authors demonstrated a significant reduction in major adverse cardiac events, and revascularization when cilostazol was added to aspirin and clopidogrel [4].

However, even if this current study did not report adverse cardiovascular outcomes, ST which was reported was not significantly different between DAPT and TAPT further supporting this analysis. In addition, this current analysis was far better since different subtypes of ST were assessed with a higher total number of participants.

A meta-analysis carried out by Zhou et al. showed no significant difference in ST with DAPT and TAPT further supporting this current analysis [20]. Additionally, another meta-analysis of randomized trials with adjusted indirect comparisons still showed no significant difference in ST with the addition of cilostazol to DAPT [21]. Major and minor bleeding events were also not increased [22].

Nevertheless, insights from a recent meta-analysis of randomized trials which aimed to show the efficacy of cilostazol on platelet reactivity and cardiovascular outcomes in patients undergoing PCI showed reduced stent thrombosis with the triple therapy [23]. The result was completely different from our current analysis. However, it should be clearly noted that in their analysis, the authors repeated data from the DECLARE trial (DECLARE-LONG, DECLARE-DM). In addition, in their analysis, bare metal stents were also included, which was not the case in this current analysis whereby only DES were used. Also, they included unpublished studies and their focus was not specifically based on ST. Our focus was centered specifically on ST and was based on published trials.

### Novelty

New features of this analysis included:

1. A high total number of participants;
2. Comparing a detailed outcome of ST (acute, sub-acute, late, definite and probable ST) in one particular paper.
3. The systematical comparison of short term and long-term ST in the general population with CAD undergoing PCI.

### Limitations

Limitations were as followed:

1. Even though all the participants were CAD patients with coronary stenting, they were different in terms of subtypes of disease and co-morbidities. A few studies reported patients with diabetes mellitus, obesity, ACS, whereas other studies involved patients with stable CAD, multi-vessel CAD, long coronary lesions, and coronary bifurcation which might affect the results.
2. More data would have significantly improved the results when assessing for definite and probable ST. However, improvement on this aspect was not possible since only few studies reported definite and probable ST among the trials which were included in this analysis.
3. Longer follow-up time periods above 5 years would have further enhanced this analysis. Nevertheless, no studies have evaluated the use of cilostazol in addition to aspirin and clopidogrel for such a longer follow up time period.
4. One observational cohort was also included among the trials.

## Conclusions

This current analysis showed no significant difference in stent thrombosis with the addition of cilostazol to the standard dual antiplatelet therapy during any follow-up time period after PCI.

## Abbreviations

DAPT: Dual antiplatelet therapy
PCI: Percutaneous coronary intervention
RCT: Randomized controlled trials
ST: Stent thrombosis
TAPT: Triple antiplatelet therapy
